# Percutaneous mechanical thrombectomy for the treatment of acute massive pulmonary embolism: case report

**DOI:** 10.1186/1477-9560-5-20

**Published:** 2007-12-18

**Authors:** Aung Myat, Arif Ahsan

**Affiliations:** 1 Cardiorespiratory Department, United Lincolnshire Hospitals NHS Trust, Pilgrim Hospital, Boston, UK; 2 Cardiology Department, Nottingham University Hospitals NHS Trust, Nottingham City Hospital Campus, Nottingham, UK

## Abstract

**Background:**

To our knowledge we report the first case of percutaneous mechanical thrombectomy used for the treatment of massive pulmonary embolism in the United Kingdom. Pulmonary embolism is a common disease process but can be difficult to diagnose. Massive pulmonary embolism presenting with profound hypotension, however, is rare. Both phenomena carry with them significant mortality. Traditionally those patients suffering haemodynamic compromise from pulmonary embolism are treated with intravenous or catheter-directed thrombolysis. When this is contraindicated surgical embolectomy or mechanical techniques via a right heart catheter are alternative options. The former is well established but the latter is less commonly utilised in clinical practice. Our aim is to highlight the effectiveness and relative safety of percutaneous mechanical thrombectomy as a therapeutic tool in massive pulmonary embolism.

**Case presentation:**

A 70 year-old gentleman presented with a 4-month history of dry cough and general malaise. Clinical examination along with routine chest radiograph confirmed a left pleural effusion which was drained. Computed tomography of the chest, abdomen and pelvis revealed a left renal mass consistent with renal cell carcinoma plus multiple metastatic subpleural nodules. Following planned thoracoscopy and pleural biopsy the patient became acutely dyspnoeic and hypotensive. Relevant investigations including computed tomography pulmonary angiogram confirmed a large saddle embolus extending in to the lobar branches of both left and right pulmonary arteries. There were several relative contraindications to thrombolysis and so the patient proceeded to have percutaneous mechanical thrombectomy with excellent results. The patient made a full recovery from the acute episode and was discharged home on warfarin with a view to planned cyto-reductive nephrectomy.

**Conclusion:**

We illustrate here that percutaneous mechanical thrombectomy can be a safe and effective method of treating massive pulmonary embolism when thrombolysis is relatively contraindicated. It may also be of use as an adjuvant therapy in those patients able to receive thrombolysis. In the future further evaluation involving a larger cohort of subjects is necessary to determine whether this treatment is superior to surgical embolectomy when thrombolysis cannot be performed.

## Background

Pulmonary embolism (PE) is a common but under-diagnosed disease process with an estimated incidence of about 60–70 per 100 000 of the general population.[1] Massive PE, however, characterised by circulatory collapse is rare and as such randomised controlled trials (RCT) on its optimal management are lacking. Indeed only one RCT comparing thrombolysis to heparin in PE with systemic hypotension exists with only 8 patients enrolled.[2] Here the trial was stopped early since all 4 patients thrombolysed survived whereas those receiving heparin died.

Despite a lack of robust data British Thoracic Society (BTS) guidelines published in 2003 clearly recommend the use of thrombolysis in massive PE; the earlier the better.[3] The BTS guidelines go on to state that if there are absolute contraindications to thrombolysis or in cases of failed thrombolysis surgical embolectomy or mechanical techniques via a right heart catheter should be considered. The former is well-established. The latter technique is lesser-known despite several successful reports from various authors. We describe how percutaneous mechanical thrombectomy can be a viable alternative to surgical embolectomy for the treatment of massive PE when thrombolysis is contraindicated. Our aim here is to highlight the availability of this treatment modality to a wider medical forum and so fully establish this technique alongside pulmonary embolectomy.

## Case presentation

A 70 year-old male presented with a 4-month history of dry cough and general malaise. He had had a right arm melanoma excised 5 months previously but otherwise had no significant medical history. Clinical examination revealed a left pleural effusion confirmed by chest radiograph which subsequently drained blood-stained fluid positive for reactive mesothelial cells and lymphocytes. Cytology was negative for malignant cells. Computed tomography (CT) of his thorax and abdomen undertaken just prior to discharge from hospital revealed a left renal mass consistent with renal cell carcinoma (RCC) and multiple bilateral subpleural nodules. Renal function was normal at this stage.

**Figure 1:**
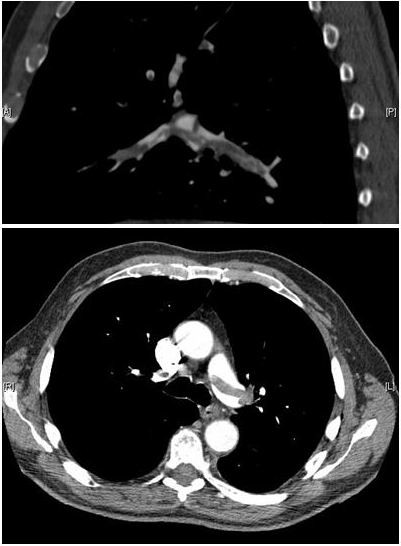
**CTPA images**. CTPA images confirming the presence of a saddle embolus and substantial thrombus burden in the lobar branches of both main pulmonary arteries.

The patient was electively re-admitted a week later for planned thoracoscopy and pleural biopsy. Biopsy confirmed the subpleural nodules to be metastatic RCC deposits. Following repeat chest drain insertion the patient collapsed some hours later and became acutely dyspnoeic with oxygen saturations of 82% on room air and a systolic blood pressure of 96 mmHg. There was no clinical evidence of lower limb deep vein thrombosis. Arterial gases confirmed type I respiratory failure and an electrocardiogram revealed a sinus tachycardia with new right bundle branch block. Subsequent CT pulmonary angiogram (CTPA) demonstrated a large saddle embolus with thrombus extending into the lobar branches of both main pulmonary arteries (Fig. 1). Massive PE was diagnosed and in view of its acute setting and the patient being in extremis the origin of the PE was not sought at this stage. Moreover a team of chest physicians; oncologists and urologists concluded that with the combination of neoplastic disease; a recent biopsy and a chest drain in-situ thrombolysis would be deleterious to the patient. Intravenous heparin was commenced whilst a cardiology opinion was sought to explore the possibility of percutaneous intervention. We as a faculty had no previous experience of using percutaneous mechanical thrombectomy (PMT) for dealing with massive PE at the time. The lead cardiologist was asked to review the patient with a view to endovascular intervention by a chest physician who wanted to exhaust all the options available. The cardiologist had remembered a case in the literature where the AngioJet system had been used to treat massive PE and from this a consensus decision was reached to pursue PMT as a definitive therapy for the patient.

**Figure 2:**
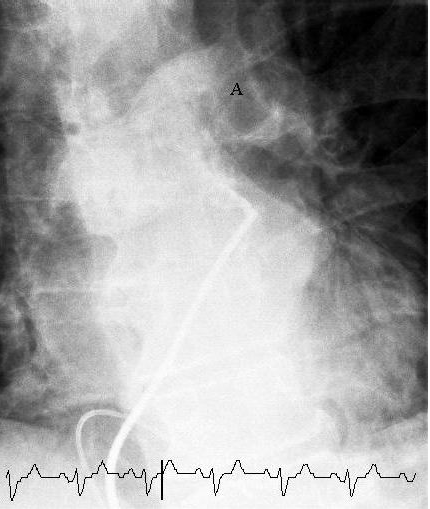
**Selective pulmonary angiogram**. Selective pulmonary angiogram revealing significant thrombus (labelled A) causing a central obstruction in the left main pulmonary artery.

We proceeded to rheolytic thrombectomy using the AngioJet system (Possis Medical, Minneapolis, MN). Access was gained via 5-French (F) and 10F sheaths into the right femoral vein. A 4F sheath was also introduced into the right femoral artery for blood pressure monitoring. We confirmed the absence of thrombus in the inferior vena cava (IVC) angiographically before proceeding with right heart catheterisation. A 0.035-inch guidewire was placed in the right ventricle over which a 5F multipurpose-1 (MP-1) diagnostic catheter was fed. Pulmonary angiography via this catheter revealed filling defects in both pulmonary arteries (Fig. 2, Additional file 1). A temporary pacing wire was then placed in the right ventricular apex via the 5F sheath to protect the patient from bradyarrhythmia.[4] Mean pulmonary artery pressure was measured at 35 mmHg.

**Figure 3:**
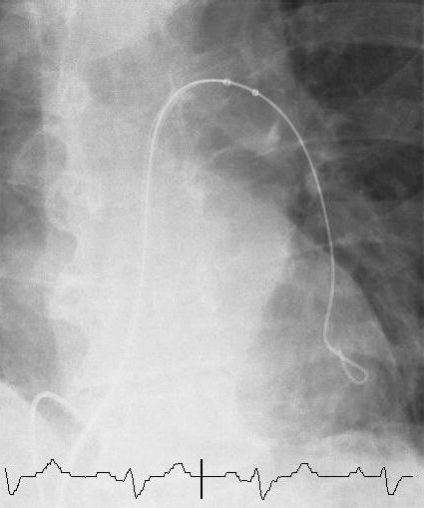
**Selective pulmonary angiogram**. Selective pulmonary angiogram showing percutaneous mechanical thrombectomy with the AngioJet catheter directed at the left main pulmonary artery.

**Figure 4:**
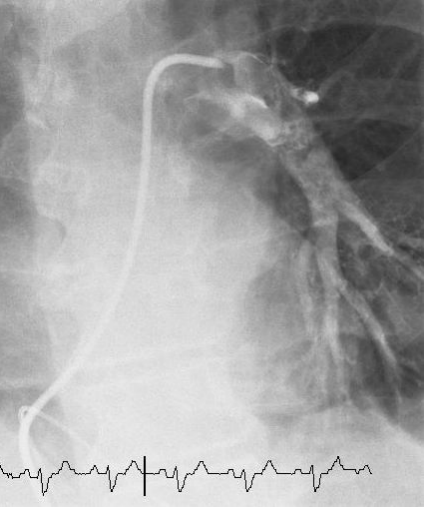
**Selective pulmonary angiogram**. Selective pulmonary angiogram revealing restoration of circulation to segmental arteries downstream of the left main pulmonary artery.

**Figure 5:**
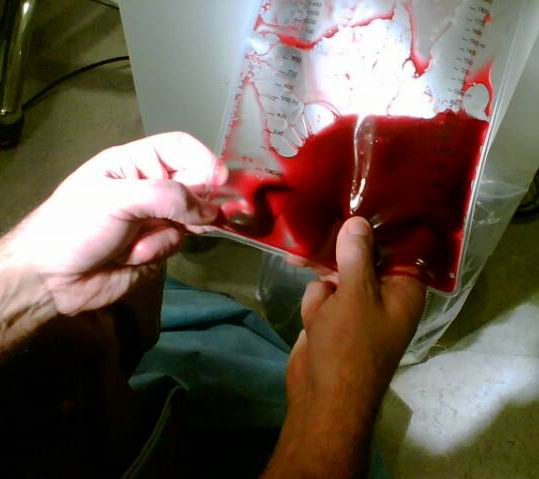
**Collection bag**. View of the collection bag containing thrombus material evacuated from the central pulmonary arterial tree using percutaneous mechanical thrombectomy via an AngioJet catheter.

Thrombectomy was performed using a 6F AngioJet Xpeedior catheter directed to both main pulmonary arteries via a 9F MP-1 catheter in the right ventricle (Fig. 3). This was undertaken through the 10F sheath in the right femoral vein. The AngioJet system was used for a total of 2 minutes and 10 seconds and selective pulmonary angiograms revealed significant reduction in thrombus burden from both left and right pulmonary arteries with significant restoration of blood flow (Fig. 4, Additional file 2). A quantitative measure of perfusion improvement was not assessed due to our relative inexperience with this procedure. Clinically, however, as perfusion returned to each main vessel the patient suffered what appeared to be a pre-syncopal episode on both occasions although all observations including heart rate, blood pressure and oxygen saturations remained within the normal range. Why this occurred remains unclear. The entire process took a total of 1 hour and 35 minutes and required 90 ml of contrast. Heart rate remained within normal range throughout. Following thrombectomy the collection bag could be seen to contain a significant amount of thrombus material (Fig. 5).

Both the temporary wire and arterial sheath were left in situ overnight and the patient remained on intravenous heparin. The chest drain was subsequently removed and the patient was off supplemental oxygen within 3 days. Ultrasound of the abdomen and pelvis on day 2 post rheolytic thrombectomy was essentially normal and showed patent IVC, hepatic, portal and renal veins. Despite this a prophylactic IVC filter was inserted in preparation for planned cyto-reductive nephrectomy in approximately six weeks and subsequent immunotherapy. Following insertion of the IVC filter the patient was warfarinised and then discharged home.

## Conclusion

PMT devices are designed to achieve rapid clearance of occlusions in large arteries, veins and bypass grafts. In massive PE the rationale behind PMT is the rapid relief of central pulmonary obstruction. PMT devices form a heterogeneous group and vary in terms of mechanism of action, efficacy, safety, and costs. Essentially, however, there are currently three available catheter thrombectomy techniques: aspiration thrombectomy, fragmentation thrombectomy, and rheolytic thrombectomy.[5] A detailed description of each technique along with their corresponding catheters is beyond the scope of this case study and has already been clearly documented in the literature.[6] Our choice of PMT device in this particular scenario owed mainly to a specific recollection of its application in massive PE from the literature as opposed to any previous experience using it.[7]

The AngioJet rheolytic thrombectomy catheter removes thrombus by use of the Bernoulli principle. It has a double-lumen shaft; one lumen for high-pressure saline delivery which loops back re-directing flow through a gap into the effluent lumen. This creates a localised low pressure area which aspirates thrombus for fragmentation into small particles. The fragmented debris then pass into a collection bag. There are disadvantages to the AngioJet system that include a significant risk of fluid overload, haemolysis, bradyarrhythmias, and the actual cost of the pump-drive set.

Rheolytic thrombectomy to treat massive PE was first described by Koning et al in 1997.[8] Despite their success the use of PMT for massive PE in clinical practice has not become widespread in the United Kingdom. This, in part, can be attributed to a lack of expertise in this area and also a lack of awareness as to the availability of this treatment modality. The former statement can be misleading, however, since this was the first time our team had attempted this procedure. In addition cases of massive PE are rare and so it is difficult to ascertain what is best practice in terms of local protocols.

The alternative to PMT is surgical embolectomy. This, however, carries with it a high mortality and can be associated with complications such as acute respiratory distress syndrome, acute renal failure, mediastinitis and severe neurological sequelae. An experienced cardiac surgical team is required and careful patient selection is necessary for mortality and morbidity benefits to be borne out.

Despite a lack of good quality randomised trials pointing to mortality benefit thrombolysis remains the mainstay of treatment for severe PE; either administered intravenously or via catheter-directed means. When this is contraindicated pulmonary catheterisation or surgical embolectomy should be considered. This recommendation remains anecdotal, however, since no trial evidence for PMT exists and no randomised trial of medical versus surgical therapy has ever taken place.[9,10] Hence the decision to go with either alternative to thrombolysis has to be made on a case-by-case basis and will depend on local availability and expertise.

We have demonstrated that PMT via a rheolytic thrombectomy catheter can be relatively straightforward in the hands of a clinician experienced in endovascular procedures. It can be a safe and effective means of clearing significant thrombus burden from the pulmonary arterial tree and can be considered a possible alternative to pulmonary embolectomy when thrombolysis is relatively or absolutely contraindicated. PMT can also be used as an adjuvant therapy prior to thrombolysis since fragmentation of the thrombus increases surface area making thrombolytic infusion more successful. In the future further evaluation involving a larger cohort of subjects is necessary to determine whether this treatment is superior to surgical embolectomy when thrombolysis cannot be performed.

## Abbreviations

PE: pulmonary embolism;

RCT: randomised controlled trial;

BTS: British Thoracic Society; 

CT: computed tomography; 

RCC: renal cell carcinoma;

CTPA: computed tomography pulmonary angiogram;

PMT: percutaneous mechanical thrombectomy;

F: French;

IVC: inferior vena cava;

MP-1: multipurpose-1.

## Competing interests

The author(s) declare that they have no competing interests.

## Authors' contributions

AM assisted with the actual procedure, helped to acquire data and images for figure presentations, drafted the manuscript and revised it critically for important intellectual content. AA conducted the actual procedure and gave a critical appraisal of the draft manuscript. All authors read and approved the final manuscript.

## Supplementary Material

Additional file 1 Selective pulmonary angiogram pre-thrombectomy. Selective pulmonary angiogram demonstrating evidence of thrombus material in the left main pulmonary artery prior to percutaneous mechanical thrombectomy. Click here for file

Additional file 2 Selective pulmonary angiogram post-thrombectomy. Selective pulmonary angiogram demonstrating restoration of blood flow in the left main pulmonary arterial tree following percutaneous mechanical thrombectomy. Click here for file
